# Noninvasive Imaging of Activated Complement in Ischemia‐Reperfusion Injury Post–Cardiac Transplant

**DOI:** 10.1111/ajt.13299

**Published:** 2015-04-23

**Authors:** E. Sharif‐Paghaleh, M. L. Yap, L. L. Meader, K. Chuamsaamarkkee, F. Kampmeier, A. Badar, R. A. Smith, S. Sacks, G. E. Mullen

**Affiliations:** ^1^ Division of Imaging and Biomedical EngineeringSchool of MedicineKing's College LondonLondonEngland; ^2^ MRC Centre for TransplantationKing's College LondonLondonEngland; ^3^ Department of ImmunologyFaculty of MedicineTehran University of Medical SciencesTehranIran

**Keywords:** animal models: murine, complement biology, diagnostic techniques and imaging, heart (allograft) function/dysfunction, ischemia reperfusion injury (IRI)

## Abstract

Ischemia‐reperfusion injury (IRI) is inevitable in solid organ transplantation, due to the transplanted organ being ischemic for prolonged periods prior to transplantation followed by reperfusion. The complement molecule C3 is present in the circulation and is also synthesized by tissue parenchyma in early response to IRI and the final stable fragment of activated C3, C3d, can be detected on injured tissue for several days post‐IRI. Complement activation post‐IRI was monitored noninvasively by single photon emission computed tomography (SPECT) and CT using ^99m^Tc‐recombinant complement receptor 2 (^99m^Tc‐rCR2) in murine models of cardiac transplantation following the induction of IRI and compared to ^99m^Tc‐rCR2 in C3^−/−^ mice or with the irrelevant protein ^99m^Tc‐prostate–specific membrane antigen antibody fragment (PSMA). Significant uptake with ^99m^Tc‐rCR2 was observed as compared to C3^−/−^ or ^99m^Tc‐PSMA. In addition, the transplanted heart to muscle ratio of ^99m^Tc‐rCR2 was significantly higher than ^99m^Tc‐PSMA or C3^−/−^. The results were confirmed by histology and autoradiography. ^99m^Tc‐rCR2 can be used for noninvasive detection of activated complement and in future may be used to quantify the severity of transplant damage due to complement activation postreperfusion.

## Abstract 2

The authors describe SPECT/CT imaging of activated complement post–ischemia reperfusion injury in mouse cardiac transplant grafts. See the supporting video at amjtransplant.com/videos.

**Abbreviations**
IRI: ischemia reperfusion injury
MRI: magnetic resonance imaging
rCR2: recombinant complement receptor 2
SPECT: single photon emission computed tomography
SPIO: superparamagnetic iron oxide
^99m^Tc‐PSMA: 99mTc‐prostate–specific membrane antigen
^99m^Tc‐rCR2: 99mTc‐recombinant complement receptor 2

## Introduction

During cardiac transplantation the heart remains ischemic for prolonged periods prior to transplantation. Upon transplantation the organ is reperfused with warm blood and this initiates a number of processes that damage the transplant. This phenomenon is called ischemia reperfusion injury (IRI) and it is one of the hurdles of any vascularized solid organ transplant 1. The cardiac graft is damaged via activation of innate components of the immune system, and the severity of these processes affects the intensity of subsequent adaptive immune responses 2.

**Figure 1:**
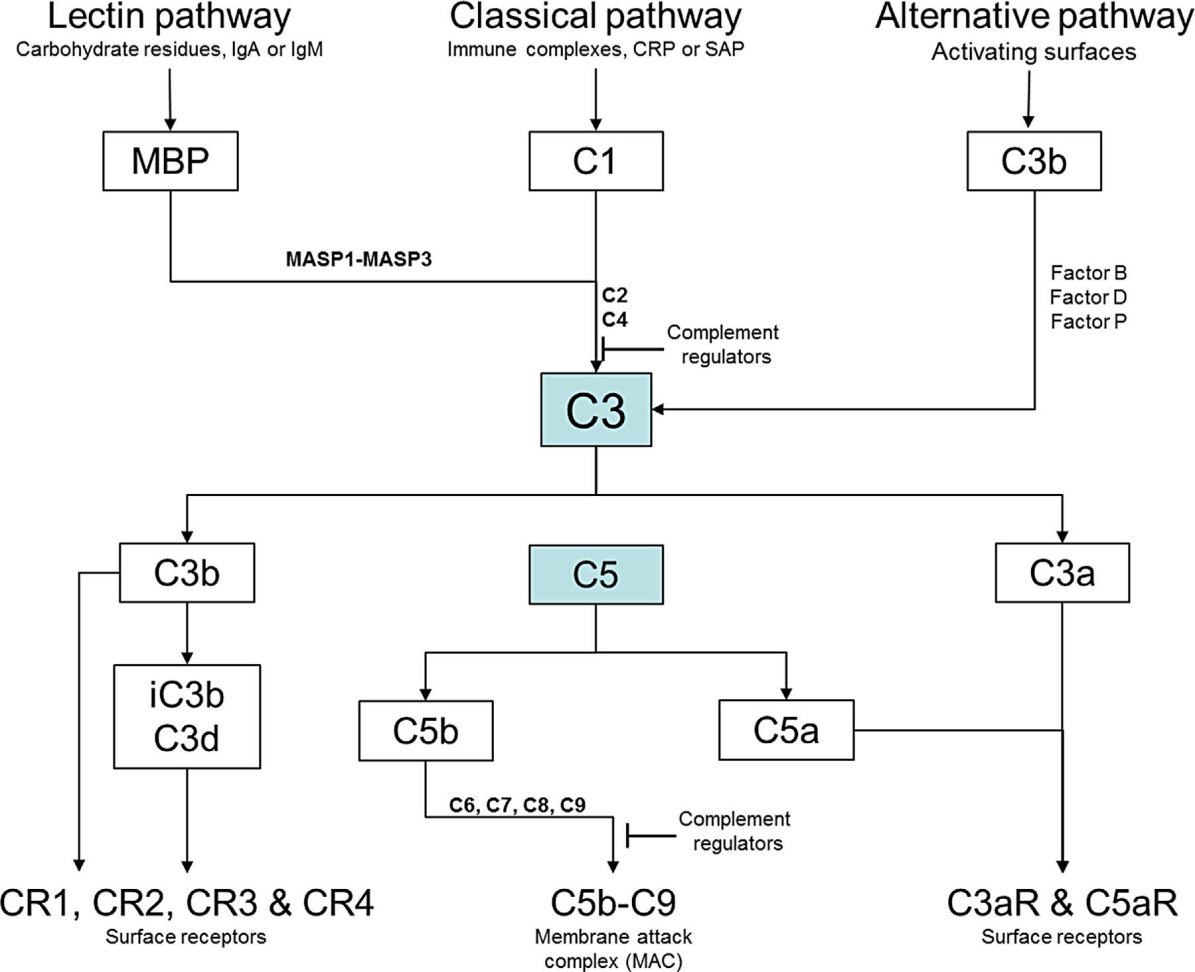
**The central complement component C3 is activated by three major pathways**. The classical pathway is triggered by immune surveillance molecules (such as IgG, IgM, and C‐reactive protein [CRP]) that are bound to the activating surface, whereas the lectin pathway is initiated by carbohydrate residues on the activating surface or by IgA and IgM, and the alternative pathway is triggered by direct binding of C3b to the activating surface. All three pathways progress to form enzyme complexes (classical or alternative convertases) that cleave either C3 (into C3a and C3b) or C5 (into C5a and C5b). C5b triggers the terminal pathway, which involves the formation of a multimeric membrane attack complex (C5b‐C9) that creates a pore in the target cell membrane. Specific cell receptors detect the soluble complement effectors (namely, C3a, C4a, and C5a) and the membrane‐bound effectors (namely, C3b and its metabolites, inactive C3b [iC3b] and C3d). Complement regulators either inhibit the C3 and C5 convertases or block the formation of C5b‐C9.

IRI has a pathophysiology consisting of at least three major factors contributing to tissue injury: *in situ* production of reactive oxygen species, activation of white blood cells (neutrophils and macrophages), and components of the activated complement (C) cascade 3. The exact process that leads to IRI is complex but complement has been shown to play an important role in IRI 2, 3, 4. Complement activation induced by IRI can involve three known pathways: the lectin (or mannose‐binding) pathway, and the alternative and classical pathways. All of these pathways converge on C3 (Figure 1). The complement protein C3 is synthesized by tissue parenchyma as an early response to tissue stress or infection 5. C3 is also abundant in the circulation, where it is mainly produced by hepatocytes. Whether synthesized locally or deposited from serum onto stressed cells, cleaved C3 attaches to the target cell surface as a C3b fragment, which is rapidly degraded to form the C3dg and C3d fragments, which remain covalently bound to the cell. Both C3 and C3b have a relatively short half‐life in serum or on the membrane and C3b degrades within minutes on the plasma membrane of the effected cell. Thereafter, C3d is relatively stable *in situ* and can be detected for several days 2. In several organ models of IRI, these covalently membrane‐bound products of C3, i.e. C3b and C3d, are associated with tissue injury 6, 7, which is caused by activation of the terminal pathway downstream of C3 cleavage (generating the complement effectors C3a, C5a, and C5b‐9, the members of attack complex, which cause inflammation and membrane injury). Therefore, C3d serves as a “footprint” of complement activation and a potential marker of tissue injury in myocardial reperfusion damage.

Complement is widely used as a biomarker for many diseases in blood, urine, and biopsy sampling 8. Also, complement has a key role in cardiac pathology whether it is ischemia‐related or not 9. However, in some cases, these assays have provided limited whole organ information, and have often yielded false‐positive or false‐negative results 10. Currently no validated techniques exist to noninvasively assess the severity of tissue injury caused by a specific pathological pathway within an affected organ soon after IRI.

Acute rejection of the transplanted organ is linked with IRI most likely as a consequence of the innate immune response enhancing antigen presentation and stimulating T cell reactivity against the donor antigen 11. Tissue damage posttransplantation can occur through cold ischemia during organ transport and warm ischemia during organ removal and subsequent reperfusion. A central aim in transplantation is to reduce the amount of reperfusion injury 12. IRI posttransplantation is associated with induction of inflammatory cytokines such as TNF‐alpha and IL‐1 13 by graft endothelium, which further triggers polymorphonuclear leukocytes, chemokines, and complement proteins 14, 15.

A noninvasive biomarker based on C3d that could identify tissue damage soon after IRI would be a powerful tool in the risk stratification of transplant patients and further guide intervention, including specific therapeutic interventions. In this regard, utilizing the complement product as a biomarker at the onset of inflammatory injury may potentially be of benefit in predicting the extent of injury, its onset, and consequences for late graft outcome. Specific complement inhibitors are now available for evaluation 16, 17 and imaging a specific molecular marker, such as C3d, could provide a more rational basis for patient risk stratification and the application and monitoring of such treatments.

Complement receptor‐2 is a natural ligand for C3d and can be adapted for imaging purposes. Thurman et al developed a CR2 molecule that is bound to superparamagnetic iron oxide (SPIO) nanoparticles and was used for renal MRI imaging in a mouse model of lupus nephritis 18, 19. These studies reported noninvasive detection of complement activation in models of kidney inflammation. We have previously developed a single‐photon emission computed tomography (SPECT) imaging agent using recombinant complement receptor 2 (rCR2); this *in vitro* specifically binds to the degraded components of activated, membrane‐bound C3 fragments, namely iC3b, C3dg, and C3d, on the surface of affected cells 20. The 15.5 kDa rCR2 radiolabelled with ^99m^Tc radioisotope was shown to only bind to cells expressing C3d *in vitro* 20. Here, we evaluated the hypothesis that rCR2 could be used for noninvasive whole organ imaging of activated complement, following IRI in mouse cardiac isografts.

## Materials and Methods

### Mice and heart transplantation

Age‐ and sex‐matched C57BL/6 (referred as BL/6, purchased from Harlan) or C57BL/6 C3^−/−^ mice (referred to C3^−/−^). The C3^−/−^ was derived by homologous recombination in embryonic stem cells and backcrossed on to BL/6 parental strain for at least 10 generations 21. Mice were maintained under specific pathogen free conditions and all experiments were conducted in accordance with national guidelines for animal care. Mice were sacrificed by dislocation of the neck at the end of the experiments.

Syngeneic heart transplantations were performed according to literature method 17. Hearts were excised, perfused with saline and stored in PBS at 4°C for 35 min prior to implantation and anastomosis time was 30 min. Donor hearts were transplanted into the abdomen of recipients by anastomosis of the recipient and donor aortas and the recipient inferior vena cava with the donor pulmonary artery. The following day and post‐iv administration of radiotracer mice were imaged using NanoSPECT‐CT. There were three groups of syngeneic transplantation: Group 1) BL/6 mice injected with ^99m^Tc‐rCR2 (n = 6); Group 2) BL/6 mice injected with an irrelevant peptide ^99m^Tc‐PSMA (prostate‐specific membrane antigen) (n = 3); and Group 3) C3^−/−^ mice injected with ^99m^Tc‐rCR2 (n = 3). As an additional control, BL/6 mice transplanted with syngeneic heart were imaged with radiolabel alone (^99m^Tc‐Tricarbonal).

### Peptide radiolabelling with ^99m^Tc

We previously reported developing a 15.5‐kDa peptide which consisted of short consensus repeats 1 and 2 of complement receptor 2 (CR2), the binding domain for C3d 22. The peptide was engineered to include the C‐terminal sequence VFPLECHHHHHH, a hexahistidine tag for site‐specific radiolabeling with ^99m^Tc(CO)_3_(OH_2_)_3_]^+^ (^99m^Tc‐Tricarbonal) 20. Radiolabelling of peptides and quality control experiments were performed as described previously 23. Briefly, ^99m^Tc pertechnetate eluted with saline from a Drytec generator (GE Healthcare, Amersham, UK) was converted to [^99m^Tc(CO)_3_(OH_2_)_3_]^+^ using a kit from Isolink (Covidien, Petten, the Netherlands). Proteins were labelled with ^99m^Tc by incubating 100 μg of rCR2 or PSMA in 100 μL of PBS pH 7.4 with an additional 350 mM NaCl and up to 600 MBq of [^99m^Tc(CO)_3_(OH_2_)_3_]^+^ in 100 μL at 37°C. Then 20 μg of radiolabelled peptides were intravenously injected via tail vein.

### *In vivo* NanoSPECT‐CT imaging

Single photon emission tomography was performed with a small‐animal NanoSPECT‐CT with silver upgrade (Mediso, Budapest, Hungary) under isofluorane anaesthesia, respiration monitoring, and a heating pad to ensure constant body temperature to 37°C. Mice were injected with 100 μL ^99m^Tc‐rCR2 (20 μg of rCR2 n = 9) or 100 μL ^99m^Tc‐PSMA (20 μg of PSMA, n = 3) intravenously via the tail vein and allowed to rest for an hour to allow distribution of radiopharmaceutical prior to imaging. The images were analyzed using the VivoQuant software (inviCRO, Boston, MA).

### *Ex vivo* organ biodistributions and histological studies

Following imaging, mice were removed from the NanoSPECT‐CT bed and sacrificed. The organs were removed, weighed, and measured for presence of ^99m^Tc. Gamma counting was carried out using a multiwell automated Wallac 1282 Compugamma Universal Gamma Counter (LKB Wallac, PerkinElmer, Cambridge, UK). Biodistribution data were expressed as the percentage injected dose/gram (%ID/g) for each organ.

For histological studies, heart samples were covered with optimal cutting temperature bedding compound (VWR Chemicals, Leuven, Belgium) and snap‐frozen in liquid nitrogen before storing at −80°C. The mid region of the hearts were sectioned (transverse axis) at 5 μm thicknesses using 5030 microtome (Bright, Cambridgeshire, UK) and placed on glass slides. The specimens were fixed by incubating with 4°C Acetone for 5 min. Slides were washed with PBS and blocked with 20% fetal bovine serum (FBS, Life Technologies, Paisley, UK) in PBS for 30 min at room temperature. Then slides were incubated with rabbit anti‐C3d (ab15981, Abcam, UK) at 1/200 dilution for 1 h at room temperature. Slides were incubated for 30 min at room temperature with secondary antibody goat anti‐rabbit Dylight 488 (ab96895, Abcam) at 1/1000 dilution. Slides were washed and DAPI mounting serum (ProLong Gold, Life Technologies, Paisley, UK) was added to co‐stain for nuclei. Images were taken using DM6000B fluorescent microscope (Leica, Wetzlar, Germany).

For autoradiography of the heart, the transplanted hearts were sectioned at 30 μm thickness using microtome and heart sections were placed on glass slides. The slides were then imaged using Instant Imager (Canberra Packard, Didcot, UK) to demonstrate localized presence of radioactivity within heart sections.

### Statistics

All analyses are reported as mean  ±  standard error of the mean (SEM). Data were analysed using GraphPad Prism Version 5 and statistical analysis were performed using one‐way analysis of variance (ANOVA) and statistical significance assigned for p values <0.05. If the one‐way ANOVA was significant, then a posthoc analysis was performed with Dunnet's test; p values of <0.05 were considered significant. Significant differences of <0.05 were labelled *, <0.01 were labelled **, and <0.001 were labelled ***.

## Results

### ^99m^Tc‐rCR2 specifically targets C3d expression in IRI via SPECT‐CT

**Figure 2:**
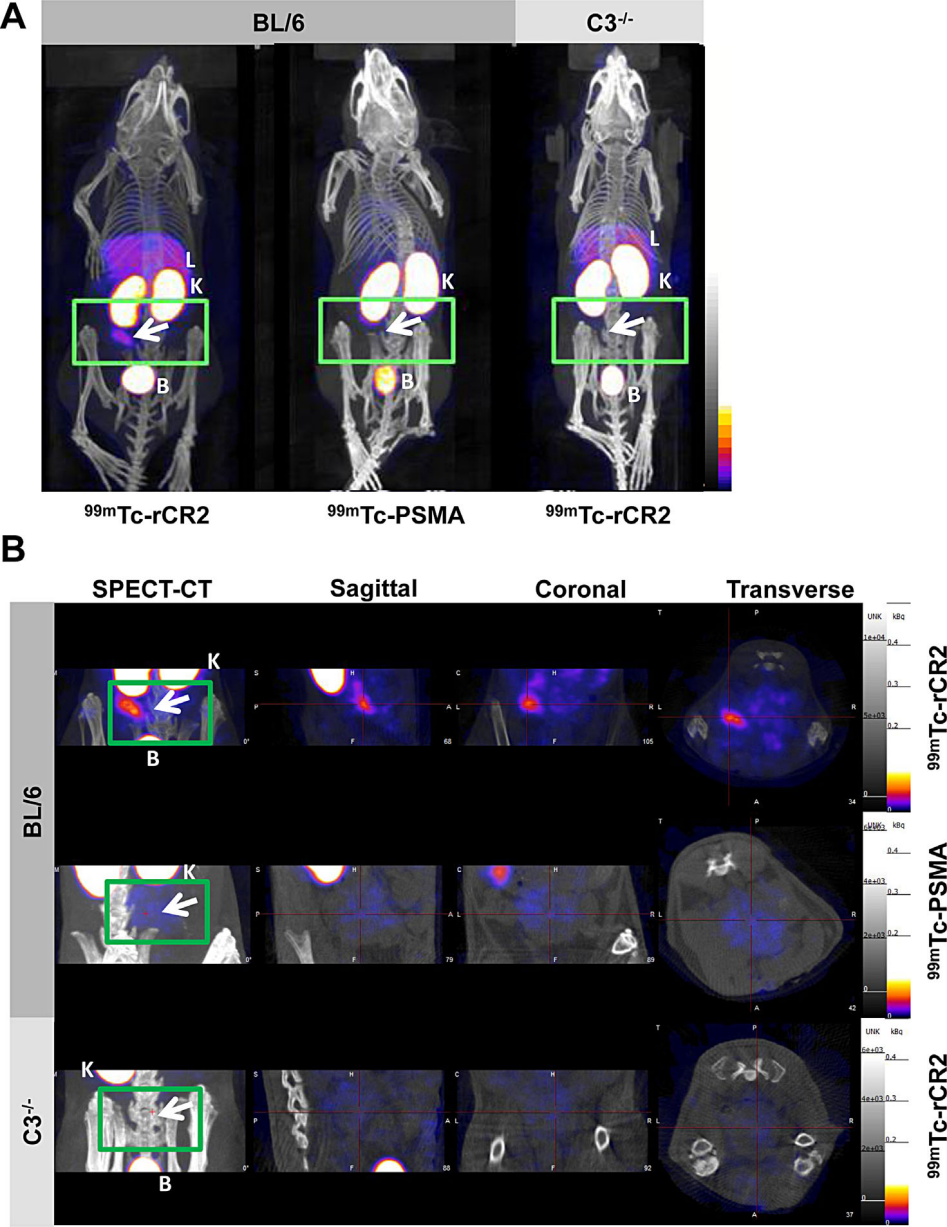
**C3d is detected by ^99m^Tc‐rCR2 imaging using NanoSPECT‐CT**. Mice that underwent heart transplantation were injected with ^99m^Tc‐rCR2 or the ^99m^Tc‐PSMA. After 1 hr, mice were imaged for 45 min via NanoSPECT‐CT. Signals were detected in the liver [L], kidneys [K], and bladder [B]. The location of the isograft heart was transplanted indicated by a green rectangle and white arrow. (A) Whole body SPECT‐CT imaging of BL/6 or C3^−/−^ mice injected with ^99m^Tc‐rCR2 or ^99m^Tc‐PSMA. (B) SPECT‐CT, sagittal, coronal, and transverse images of the region of the heart transplant. SPECT, single photon emission computed tomography; CT, computed tomography; PSMA, prostate–specific membrane antigen; CR, complement receptor.

**Figure 3:**
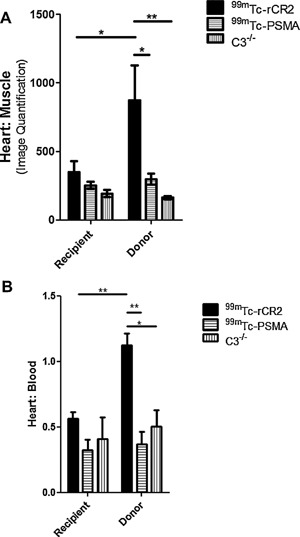
**Biodistribution studies confirmed presence of ^99m^Tc‐rCR2 in the donor hearts**. (A) Quantification of SPECT‐CT images for levels of radioactivity in the heart transplant was carried out. The heart signals were corrected to muscle signals. (B) After imaging studies, mice were culled and the recipient and donor hearts were removed, weighed, and *ex vivo* biodistribution was performed. p values <0.05 and <0.01 are labelled as ^*^ and ^**^, respectively (^99m^Tc‐rCR2 n = 6, ^99m^Tc‐PSMA n = 3, and C3^−/−^ n = 3).

In order to determine if we could detect complement activation post‐IRI, we investigated whether radiolabeled rCR2 could be used to noninvasively image activated complement post‐IRI in a cardiac transplant model. We performed heterotopic isograft heart transplantation in mice with a fixed cold and warm ischemia times that were found to induce C3d deposition 17. The day after transplantation, we intravenously injected mice with 50 MBq of ^99m^Tc‐rCR2 in C57BL/6 (Group 1) or C3^−/−^ (Group 3) and ^99m^Tc‐PSMA in C57BL/6 (Group 2). They were then imaged for 45 min, using a NanoSPECT‐CT Plus, 1 h postinjection. Uptake of ^99m^Tc‐rCR2 was observed in the transplanted heart (indicated by white arrows in Figures 2A and B and S1A) but was not observed in ^99m^Tc‐PSMA (Group 2) or C3^−/−^ (Group 3). We performed image quantification of the SPECT/CT images and observed a significant uptake in Group 1 (872.5 ± 255.3) compared to Group 2 (298.4 ± 38.8) and 3 (163.2 ± 12.7) (Figure 3A) (p = 0.030). Also, the transplant to muscle ratio of Group 1 was significantly higher than that of Group 2 (p = 0.027) or C3^−/−^ Group 3 (p = 0.029).

All three groups demonstrated high uptake in the kidney and bladder due to the nonspecific retention and clearance of the radiolabeled protein into the urine (Figure 2A and video in Figure S1A). In addition, the groups that were injected with ^99m^Tc‐rCR2 also showed some uptake in the liver. Overall, this demonstrates that ^99m^Tc‐rCR2 can detect complement activation and C3d expression post‐IRI in the transplanted heart of BL/6 mice and not in the C3^−/−^ or in BL/6 mice imaged with ^99m^Tc‐PSMA.

### *Ex vivo* biodistribution studies of ^99m^Tc confirmed *in vivo* SPECT‐CT images

To confirm specific uptake of ^99m^Tc‐rCR2 in the transplanted hearts, mice were euthanized after imaging and organs removed for gamma counting and histology. Organs were weighed and measured for presence of radioactivity and % Injected Dose (ID) per gram calculated (Figures 3B and S2). Group 1 heart transplants demonstrated significantly higher uptake (1.12 ± 0.16) as compared to C3^−/−^ (0.51 ± 0.17) (p = 0.012) or ^99m^Tc‐PSMA (0.37 ± 0.17) (p = 0.002) transplants. No significant uptake above background (defined as activity in the blood) was observed in the native hearts and there was no significant difference observed between the three groups (p > 0.05). Both *ex vivo* biodistribution and noninvasive quantification of images confirmed what was observed in the noninvasive SPECT‐CT images of the transplanted heart (Figures 2 and S1).

### Immunohistological studies confirmed C3d expression in the transplanted heart

**Figure 4:**
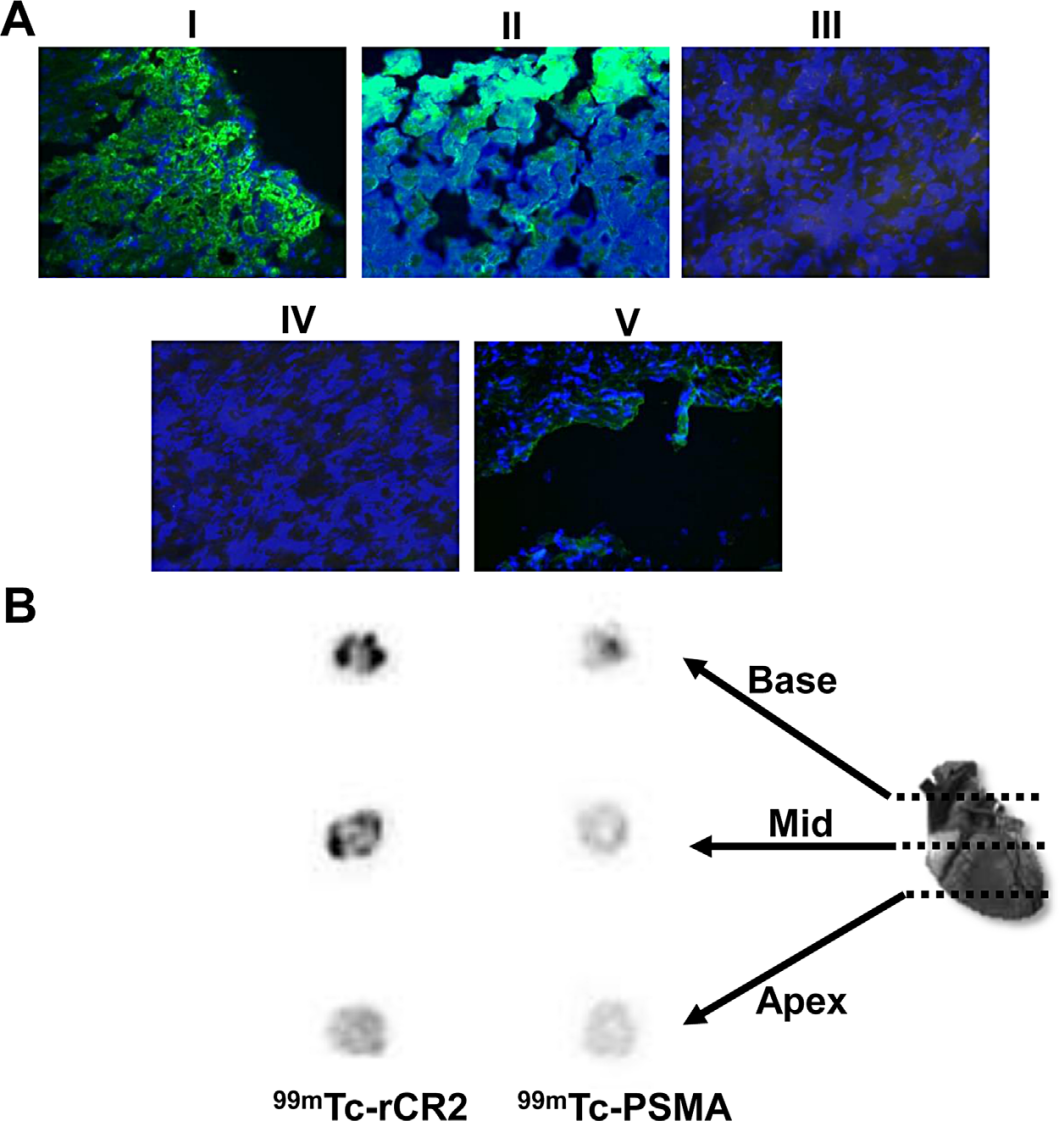
**Histological studies demonstrating C3d expression on the transplanted heart**. (A) After imaging studies, transplanted hearts were removed, sectioned, and stained with anti‐C3d (green) and DAPI (blue). Heart sections were from mid region of the transplanted heart of BL/6 mice injected with ^99m^Tc‐rCR2 (I), ^99m^Tc‐PSMA (II), and C3^−/−^ mice injected with ^99m^Tc‐rCR2 (III). Control for staining was transplant heart sections stained with secondary antibody alone (IV) and C3d staining in the native recipient heart of mice injected with ^99m^Tc‐rCR2 (V). All images were acquired at 40 × magnification. (B) After imaging, transplanted hearts from BL/6 mice injected with ^99m^Tc‐rCR2 or ^99m^Tc‐PSMA were sectioned and presence of radioactivity within heart tissue was studied using autoradiography. A representative section of the base, mid and apex of the heart is shown. Darker regions represent areas with higher amounts of radioactivity. PSMA, prostate–specific membrane antigen; CR, complement receptor.

To investigate whether the significant uptake of ^99m^Tc‐rCR2 observed in the transplanted heart images (Figures 2 and S1) correlated with the presence or absence of C3d expression, histological analysis was performed. The transplanted hearts were stained for C3d (green) and nucleus (DAPI, blue) (Figure 4A). The expression of C3d was observed in the transplanted heart of both ^99m^Tc‐rCR2 and^99m^Tc‐PSMA groups but not C3^−/−^. This confirmed that the uptake observed in the transplanted heart of BL/6 mice injected with ^99m^Tc‐rCR2 (Group 1) was associated with tissue C3d. It also demonstrated that C3d was present in Group 2 transplants, where as no imaging signal was associated with the irrelevant peptide. As expected, the C3^−/−^ transplanted hearts (Group 3) did not show any C3d expression. To identify where the ^99m^Tc‐rCR2 was located within the transplanted heart, sections of the base, mid, and apex of transplanted hearts from all three groups were assessed for presence of radioactivity by autoradiography. Heart sections of mice injected with ^99m^Tc‐rCR2 (1.166 ± 0.039) showed significantly higher levels of radioactivity compared to ^99m^Tc‐PSMA (1.064 ± 0.019) injected mice (p = 0.0003). Within the transplanted heart of mice injected with ^99m^Tc‐rCR2, a higher signal was observed in the base of the heart than the mid or apex of the heart. Overall, the histological and autoradiography data presented here illustrate that ^99m^Tc‐rCR2 can specifically detect C3d expression post‐IRI in the transplanted hearts.

## Discussion

Detection of complement in ischemia reperfusion injury has been reported in numerous preclinical and clinical studies 23, 24. Here, we aimed to develop a noninvasive tool to detect activate complement; however, the molecular specificity of this assessment has been uncertain due to variability in readouts of complement activation 10. We used rCR2 peptide to detect the terminal quasi stable product of activated complement, namely C3d. Firstly, CR2 is a specific receptor for complement activation and binds to the terminal complement product, C3d 20. Secondly, the incorporation of a hexahistidine tag in rCR2 peptide allows for site‐specific radiolabeling without the fear of disrupting the CR2–C3d binding site 20. We studied complement activation induced by IRI in a heart transplant model. We demonstrated that radiolabeled rCR2 could be u­sed to detect complement activation and, in particular, C3d expression on the transplanted heart, which had undergone 35 min of cold ischemia followed by 24 h reperfusion. We managed to noninvasively detect C3d expression on the transplanted heart and confirmed our findings through *ex vivo* biodistribution and histological studies.

There are many reports of the involvement of complement in transplant rejection (reviewed in 25). It has been demonstrated that pretransplant complement expression on the graft correlates to early and late graft function 26. Therefore, a long‐term objective of ^99m^Tc‐rCR2 would be to quantify complement activation in the transplant as early as possible prior to a rejection event and also monitor treatment. This would be an important step forward to image graft inflammation noninvasively as biopsies of the graft are not without risk. Moreover, anti‐complement therapies have an emerging role in transplant patients 27 and ^99m^Tc‐rCR2 could be used as an interim measure of response to treatment.

The molecular tracer described here, ^99m^Tc‐rCR2, could also be applied to other solid organ transplants and would be of wider interest in diseases that trigger the complement system, such as rheumatoid arthritis (28), systemic lupus erythematosus (29), autoimmune myocarditis 30, and IRI 24, which itself can be involved in many diseases such as myocardial infarction..

In summary, we demonstrated that ^99m^Tc‐rCR2 can be used to noninvasively image *in situ* deposition of C3d on transplanted hearts as a result of IRI. This could be a promising method for monitoring the inflammatory status of transplanted organs and specifically to assess the damage of the transplanted organ as a result of IRI. Moreover, ^99m^Tc‐rCR2 coupled with SPECT imaging could have great application as a noninvasive monitoring agent for diseases that involve complement.

## Author Contributions

ES‐P, MLY, RAS, SS, and GEM designed research; ES‐P, MLY, LLM, KC, AB, and FK performed research; ES‐P, MLY, SS, and GEM analysed data; and ES‐P, RAS, SS, and GEM wrote the paper.

## Disclosure

The authors of this manuscript have no conflicts of interest to disclose as described by the *American Journal of Transplantation*.

## Supporting information


**Figure S1: SPECT‐CT movie of imaging complement protein by ^99m^Tc‐rCR2.** Mice that underwent heart transplantation were injected with ^99m^Tc‐rCR2 or the control peptide ^99m^Tc‐PSMA or ^99m^Tc‐Tricarbonal. After 1 h, mice were imaged using NanoSPECT‐CT. The region where the isograft heart was transplanted is indicated with a green rectangle. (A) Whole‐body SPECT‐CT imaging of BL/6 or C3^−/−^ mice injected with ^99m^Tc‐rCR2 or ^99m^Tc‐PSMA or ^99m^Tc‐Tricarbonal. (B) SPECT‐CT movies of the abdomen of mice where the heart was transplanted.
**Figure S2: *Ex vivo* organ biodistribution study of presence of radiotracer in organs.** After imaging studies, mice were culled and organs were removed and weighed. Then organs were measured for presence of ^99m^Tc. Biodistribution data were expressed as the percentage injected dose/gram (%ID/g) for each organ.
